# General practitioner consultations for mental health reasons prior to and following bereavement by suicide

**DOI:** 10.1007/s00127-023-02607-9

**Published:** 2024-02-06

**Authors:** Sissel Marguerite Bélanger, Lars Johan Hauge, Anne Reneflot, Carine Øien-Ødegaard, Solveig Glestad Christiansen, Per Magnus, Kim Stene-Larsen

**Affiliations:** 1 Department of Mental Health and Suicide, Norwegian Institute of Public Health, Oslo, Norway; 2 Department of Alcohol, Tobacco and Drugs, Norwegian Institute of Public Health, Oslo, Norway; 3 Centre for Fertility and Health, Norwegian Institute of Public Health, Oslo, Norway

**Keywords:** Suicide, Suicide bereavement, Primary health care, General practitioner

## Abstract

**Purpose:**

Prior research has shown that the majority of those bereaved by suicide express a need for mental health care services. However, there is a lack of knowledge about these individuals’ use of primary health care. The objective of our study was to estimate the association between suicide bereavement and general practitioner (GP) consultations for mental health reasons.

**Methods:**

A population-wide, register-based cohort study identifying 25,580 individuals bereaved by suicide. Estimations of increases in consultation rate were modeled through individual fixed-effects linear analyses adjusted for age and time-period.

**Results:**

Overall, 35% of those bereaved by suicide had a GP consultation for mental health reasons during the first 1–2 months, and 53% after two years. In the month immediately after bereavement by suicide, there was a large increase in the consultation rate with a GP for mental health reasons. In the months that followed, the consultation rate gradually decreased. One year after bereavement, the consultation rate stabilized at a somewhat higher level than before the death. The increase in consultation rate was evident across all kinship groups, and the increase was greatest for partners and smallest for siblings. Women had more contact with the GP before the suicide and a greater increase in contact than men.

**Conclusion:**

Our findings suggest that many of those bereaved by suicide seek assistance from primary health care, and that some are in need of prolonged follow-up from the GP. Health governments should be aware of this and seek to strengthen the GPs knowledge of the needs and challenges associated with this patient group. Measures should also be taken to remove barriers to contact the health care system, especially for men and bereaved siblings.

**Supplementary Information:**

The online version contains supplementary material available at 10.1007/s00127-023-02607-9.

## Background

Losing a loved one to suicide is an extremely stressful event affecting a considerable number of individuals [1–4]. Suicide bereavement is associated with an increased risk of mental health problems [5] and a majority indicate a need for professional help [6, 7]. General practitioners (GPs) are well suited to meet the needs of most of these [8, 9], and also have an important role in detecting and referring those in need of specialized health care. The GP plays a key role in the Norwegian health care system. Norway has universal health care that is affordable for all, making private health care less common. All residents have the right to a regular GP and the GP acts as a gatekeeper for more specialized health care.

Despite the important role of GPs in the follow-up of suicide bereaved, little is currently known about the timing and extent of contact suicide bereaved have with this part of the health care system [8]. A limited number of studies have examined this but show contrasting findings and are prone to selection bias [6, 7, 10, 11]. To our knowledge there are only two population-based studies of contact with a GP among suicide bereaved. The first study investigated differences among suicide bereaved spouses compared to spouses bereaved by other causes of death and the general population, they found a reduced likelihood of any-cause GP consultations among women compared to the general population, and among men when compared to other bereaved, within five years after the death [12]. The second study examined contact with GPs for mental health reasons among adolescents in the years before and after the suicide of a parent [13] and showed an increase in consultations in the quarter the parent died, this risk remained elevated in subsequent years. This study provided valuable information on how the contact rates changed over time following parental suicide. This is of importance as those bereaved by suicide emphasize the need for access to support immediately after the loss [14] and early intervention might prevent negative long-term consequences [15]. Despite this, one study found that those bereaved by suicide were more likely to perceive delays in receiving formal support than those bereaved by other causes [16]. Including time before bereavement is also informative as several studies have shown that those bereaved by suicide differ from the general population already before the loss, regarding factors such as mental health and socioeconomic status [5, 17, 18].

Negative effects of suicide bereavement vary according to the relationship to the deceased [5], and this is likely the case for health care use as well. Gender of the bereaved is also a potential moderator of the association between suicide bereavement and health care use, as men have been shown to use primary health care [19] and suicide survivor support groups [20] to a lesser extent than women.

In the current study, we aim to explore the impact of suicide bereavement on GP consultations for mental health reasons in the years before and after the loss with the use of descriptive statistics and within-individual models that control for pre-existing differences. Other groups of bereaved will be included for comparison. A secondary aim of the current study is to examine differences between genders and types of kinships to the deceased, potentially uncovering how these factors relate to GP consultations for mental health reasons before and after suicide bereavement.

## Methods

In this study, we use data covering the entire Norwegian population from January 1st 2006 to December 31st 2020. With the use of the unique personal identifier assigned to all Norwegian residents we linked data from the Norwegian Cause of Death Registry (NCDR), the Norwegian Population Registry (DSF), and the Norwegian Control and Payment of Health Reimbursements Database (KUHR). Participants were included at the start of the study period or when they turned 20, whichever came last. Participants were censored out when they turned 80, at the second death if they experienced bereavement by suicide more than once since 1996, if they emigrated, or at the end of the observation period.

The study is part of a project funded by the Research Council of Norway (Treatpath-project, project number 288731). The study received ethical approval from the Regional Committee for Medical and Health Research Ethics (2019/321).

### Variables and data sources

Information on date and cause of death was retrieved from the NCDR. We coded cause of death as either suicide (ICD-10 codes X60-X84 and Y87.0), other external deaths (ICD-10 codes from chapter V, W, X and Y except X60-X84 and Y87.0), or other (all remaining). DSF contains information on parent–offspring relations and spouses linked through their unique personal identifiers. We were also provided with data on cohabitants that are estimated based on registry data by statistics Norway [21]. This was used to identify those who were bereaved and the kinship to the deceased. Kinship was categorized as partner (spouses and cohabitants), offspring, parent, or sibling. Birthyear and legal gender of the bereaved and deceased were also obtained from DSF.

The outcome variable was monthly number of consultations with a GP for mental health symptoms or diagnoses (ICPC-2 codes P01-P99). This information was obtained from KUHR which contains information on all billable contact with GPs. In Norway, all medical contacts require a diagnostic code to be valid for reimbursement from the state, and because of this more or less all contact with GPs are registered with at least one diagnostic code. The outcome variable was capped at five consultations to handle outliers (affecting < 0.01% of person-months).

### Statistical analyses

To examine the effect of suicide bereavement on GP consultations for mental health reasons we estimated individual fixed-effects linear models. We constructed person-months, following individuals for a maximum of 180 months (15 years), resulting in an unbalanced sample. The analyses were carried out in Stata version 17.0 using the xtreg-command with robust standard errors clustered at the individual level. To examine how associations between bereavement and consultation rate varied over time we included a variable indicating time before/after the event up to two years. More specifically we used a vector of duration dummies for one-month categories, with non-bereaved controls having zero on all of these. Two years prior to bereavement was chosen as the reference category.

The prevalence of GP consultations for mental health reasons is believed to change both over time and with age. Non-bereaved controls were, therefore, included in the model to control for both time-trends and age effects with the underlying assumption that these effects are similar in the control group and among suicide bereaved. Period was included as a set of dummies indicating one-month categories. This allowed us to control for both seasonal effects within each year as well as time-trend effects. Dummy variables indicating one year age categories ranging from 20 to 79 years were included to control for age.

Individual fixed effects models handle confounding from all observed and unobserved time-constant factors as they compare individuals with themselves over time. This means that our model considers underlying static differences between groups that affect health care use. But this also means that such factors cannot be included in the model, as temporal variation is required for the estimation of coefficients. To examine how cause of death, gender and kinship to the deceased might interact with GP consultations for mental health reasons after bereavement we conducted a series of model estimations stratified according to these factors. To check if differences between these groups were statistically significant we also ran models including cause of death, gender and kinship as interaction terms.

## Results

We identified 25,580 individuals who had been bereaved by the suicide of a partner, parent, offspring or sibling during the study period. There were 8,685 registered suicides in Norway during the study period, resulting in an average of 2.9 bereaved persons per suicide death that were identifiable in registers. Descriptive statistics for non-bereaved controls and bereaved groups are included in Table 1 and show group differences regarding gender, age of both the bereaved and deceased, and kinship to the deceased. Among those bereaved by suicide those losing an offspring were the oldest (*m* = 60.8, SD = 10.5), those losing a parent were the youngest (*m* = 35.8, SD = 11.1), with those losing a partner (*m* = 50.1, SD = 14.1) and a sibling (*m* = 45.9, SD = 13.6) in between.

**Table 1:** Descriptives

	Non-bereaved controls (*n* = 3,486,363)	Bereaved by other causes (*n* = 1,194, 070)	Bereaved by external causes (*n* = 57,593)	Bereaved by suicide (*n* = 25,580)	Total (*n* = 4,763,606)
Gender *n* (%)					
Female	1,675,016 (48.0)	612,507 (51.3)	30,579 (53.1)	14,773 (57.7)	2,332,875 (49.0)
Male	1,811,347 (52.0)	581,563 (48.7)	27,014 (46.9)	10,807 (42.3)	2,430,731 (51.0)
Age* (years) mean (SD)	43.37 (17.5)	53.3 (12.3)	50.8 (14.0)	47.1 (15.2)	46.3 (16.0)
Age (years) of the deceased mean (SD)		76.7 (13.8)	66.4 (22.7)	48.2 (16.5)	
Kinship to the deceased *n* (%)**					
Partner		154,237 (12.9)	5824 (10.1)	2820 (11.0)	
Offspring		26,983 (2.3)	6565 (11.4)	6164 (24.1)	
Parent		884,618 (74.1)	33,457 (58.1)	6406 (25.0)	
Sibling		128,232 (10.7)	11,747 (20.4)	10,190 (39.8)	

*Overall person-months for non-bereaved controls and total, at time of bereavement for those bereaved

**Describes the person who has died from the perspective of the bereaved

**Fig. 1:**
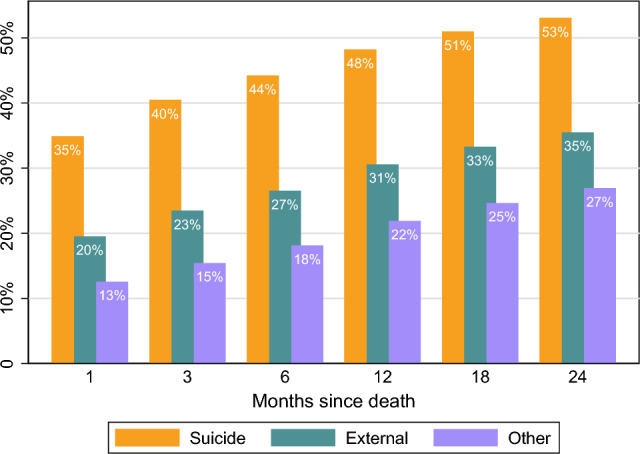
Cumulative proportion in contact with GP for mental health reasons following bereavement from suicide deaths, external deaths and other deaths

Figure 1 shows the cumulative proportion of bereaved that have had at least one GP consultation for mental health reasons since their loss. In the month after the loss, 35% of those bereaved by suicide have already had a consultation, which is 15 and 22 percentage points higher than those bereaved by external and other causes, respectively. During the first two years after a suicide loss around half of the bereaved have been in contact with the GP for mental health reasons.

**Fig. 2:**
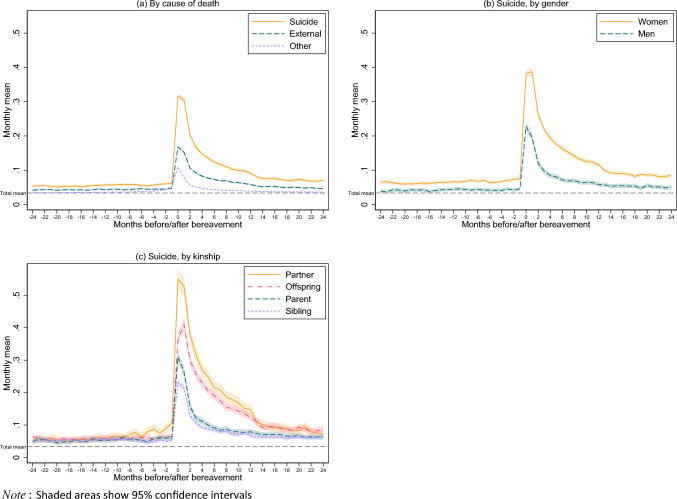
Mean number of GP consultations for mental health reasons per month by cause of death, gender, and kinship

Figure 2 shows the mean number of monthly GP consultations for mental health reasons by cause of death (panel a), gender (panel b) and kinship (panel c). The total mean for the entire sample (bereaved and non-bereaved) across all months was 0.03 consultations per month (gray dashed line). In the two years prior to bereavement, the mean number of consultations for those bereaved by suicide was 0.05–0.06 consultations per month (panel a). In other words, we observe a difference between those bereaved by suicide and the general population, already before the loss. Those bereaved by other external causes also exhibit a somewhat heightened mean before the loss, but not as much as those bereaved by suicide. Those bereaved by other causes have a mean before the loss that is very similar to that of the general population. There is a clear gender difference in pre-bereavement mean of monthly GP consultations for mental health reasons for those bereaved by suicide (panel b), with women having a higher use and the mean for men being slightly above the population mean (*m* = 0.06–0.07 and 0.04, respectively). With regards to kinship (panel c), all groups show a similar heightened mean number of GP mental health consultations before the death (*m* = 0.04–0.06).

In general, we observed a sharp increase in GP consultations for mental health reasons when the death occurred for all types of loss (panel a), with the mean for those bereaved by suicide being the highest (*m* = 0.32). The peak monthly mean for women was 0.39, which was substantially higher than that of suicide bereaved men (*m* = 0.23) (panel b). Regarding kinship to the deceased, partners bereaved by suicide had the highest peak (*m* = 0.55), followed by those who lost an offspring (*m* = 0.36), a parent (*m* = 0.31), and a sibling (*m* = 0.23) (panel c). In the months following the death, the monthly mean decreases for all groups. For the suicide bereaved group the mean consultation rate seems to stabilize at around 0.07 from 16 months after the death (panel a). Among suicide bereaved women the mean stabilizes at around 0.08, and at 0.05 for men (panel b). Suicide bereaved partners and offspring stabilize at around 0.08–0.09, and those who lost a parent or sibling at 0.06–0.07 (panel c).

**Fig. 3:**
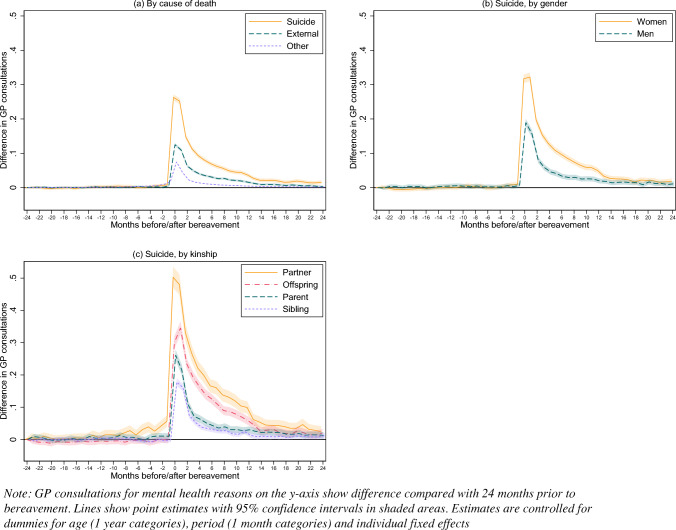
Effects of time from bereavement on monthly number of GP mental health consultations by cause of death, gender and kinship

The main regression results are presented in Fig. 3 in the form of difference in monthly GP consultations for mental health reasons compared to 24 months prior to bereavement. Results are also presented in Supplementary Tables S1, S2, S3 in supplementary material. These are controlled for age and time-period and take into account time invariant pre-existing differences between groups. There is clearly a larger increase of frequency of GP consultations for mental health reasons when the deceased died by suicide (*b*_0_ = 0.26, 95% CI 0.25–0.27) compared to both external deaths (*b*_0_ = 0.13, 95% CI 0.12–0.13) and other deaths (*b*_0_ = 0.08, 95% CI 0.07–0.08) (panel a). There is no significant (0.05-level) change in the consultation rate until the last month before the death (time = − 1) for those bereaved by suicide. The change (coefficients) stays significant until the last month included in the analysis (time = 24) and is also significantly different from that of the external and other death groups.

Gender stratified analyses of those bereaved by suicide (panel b) show a considerable gender difference around the time of bereavement, with women having about 50% higher increase in consultations compared to men (*b*_0_ = 0.32, 95% CI 0.30–0.33 and *b*_0_ = 0.19, 95% CI 0.18–0.20, respectively). The interaction between gender and time before/after bereavement stays statistically significant throughout the first year after bereavement.

Examining kinship among the suicide bereaved (panel c), the highest increase is found among those losing a partner (*b*_0_ = 0.50, 95% CI 0.47–0.54), followed by those losing an offspring (*b*_0_ = 0.30, 95% CI 0.28–0.32), a parent (*b*_0_ = 0.26, 95% CI 0.25–0.28), and a sibling (*b*_0_ = 0.18, 95% CI 0.17–0.19). The increase in consultations rates peaks after the first one to two months following the loss for all these groups, and then declines again in the following months with a sharper decrease for those losing a parent or a sibling. Partners are the only ones showing a statistically significant increase in monthly consultations rates prior to bereavement, starting at five months before. All groups have a significantly heightened consultation rate in the first year following bereavement. The rate stays significantly different for partners throughout all time points, for those losing an offspring and parent the change in rate is nonsignificant in the last 2–3 months of the analyses, while siblings return to non-significant levels after the first year (Fig. 3).

## Discussion

We aimed to explore the impact of suicide bereavement on GP consultations for mental health reasons in the years before and after loss. The results show that losing someone to suicide leads to a significant increase in GP consultations for mental health reasons. The size of this effect depends on the kinship to the deceased and the gender of the bereaved, with women exhibiting a larger increase than men and bereaved partners having the largest increase followed by those losing an offspring, parent and sibling.

Our finding that those bereaved by suicide had a higher contact rate with their GP for mental health reasons compared to both the general population and those bereaved by other types of deaths is in line with previous studies finding pre-bereavement differences [5]. Reasons for this might be both genetic and/or environmental risk factors for mental health problems shared with the deceased and assortative mating for partners. The strain of having a suicide threatened relative might also affect mental health, thus one could expect an increasing contact rate as the time of death approaches. We did not find such a pattern, except for partners. One plausible explanation for this is that partners are often the ones closest to the person dying and might experience more of the strain of taking care of a person in crisis.

Compared with those bereaved by other causes, the impact of losing a relative to suicide on GP consultations for mental health reasons is much stronger. This is also reflected in the proportion having had at least one consultation after the death being highest in the suicide group at all time points. Caution is warranted when comparing these groups as they may differ in important aspects. For instance, the age of the deceased is higher in the other groups, and the distribution of kinship types varies. These factors probably contribute to the differences in impact. In any case, our findings do not indicate that those bereaved by suicide are less in contact with their GP for mental health reasons than other bereaved. Regarding the time at which help is received, one study has shown bereaved by suicide were more likely to receive formal help at a delayed time compared to other bereaved [16]. Such a tendency was not found in the current study as the peak consultation rate was in the 0–1 months following the death and that around one third had seen their GP for mental health reasons at least once at this point of time. As time passed after the death, we observed a decrease in consultation rate and a stabilization at a somewhat higher level than pre-bereavement. It is natural that the contact rate is highest right after the death and might reflect that many need assistance to deal with the initial shock such as sick leave, prescriptions and information. Many might also need a referral to specialized health care, and one would expect contact with the GP to decline when patients receive treatment elsewhere.

Two previous studies assessing the needs of those bereaved by suicide showed that a large majority (around 90%) indicated a need for professional help, while around half the sample indicated that they had received formal help [6, 7]. The latter is comparable to the proportion of those bereaved by suicide that have been in contact with their GP for mental health reasons in the current study. This might point to an under-usage of primary health care after suicide bereavement. On the other hand, the mentioned studies are prone to selection bias and might overrepresent bereaved keenly interested in receiving treatment and services. The GP is only one of several sources of formal support available after suicide bereavement. As one has to go through the GP to access specialized psychiatric health care, most of those receiving this type of help will first have seen a GP. Private health care providers are an option for those who can afford it, but the number of private health services is relatively low in Norway [22]. Another important source of support is from NGOs offering support groups or counselling [8], although evidence from a Danish study indicates that a minority of those bereaved by suicide sought this kind of help [20]. We did not have information on alternative sources of support, and receiving help elsewhere might be a reason for not contacting a GP after bereavement. However, rather than seeing different sources of support as alternatives, previous research points out that those bereaved by suicide desire a range of assistance forms [7, 8]. It could also be the case that those accessing one type of support are more likely to also get support elsewhere. One recent report found this to be the case with an association between informal and formal support, open-ended qualitative questions pointed towards informal support from friends and family being an important reason for seeking professional help [23].

In this study, men had a lower rate of GP consultations for mental health reasons than women in the years leading up to the death, as would be expected [19]. This gender difference was amplified when the death occurred and remained high in the first year. These findings support the notion that gender is an important determinant of how those bereaved by suicide will respond to the loss [24]. It might be that men engage in different coping styles than women and, therefore, to a lesser degree rely on help from the health care system. On the other hand, men and women are shown to have a similar increase in risk of mental disease and suicide after suicide bereavement [12, 24]. Considering this, it seems likely that at least some of the large gender differences can be ascribed to an under-usage of primary health care among suicide bereaved men.

Regarding the kinship to the deceased, we found considerable differences between the four groups of suicide bereaved. When the death occurred, all groups exhibited a clear increase in the rate of GP consultations due to mental health reasons. The increase was highest for partners, maybe reflecting that this group undergo the largest changes in their life situation because of the death. Although we controlled for age, the difference seen between kinship groups might still be influenced by age differences between the groups that can lead to differences in occupational status and the need for sick leave. Siblings exhibited the lowest increase. Losing a sibling to suicide has received less attention compared to other relationships and suicide bereaved siblings have been referred to as “the forgotten bereaved” [25]. The experiences of suicide bereaved siblings vary, with some having difficulties reaching out for help because of overwhelming grief, while others do not experience a need for professional help [26].

The use of registry data entails some important limitations. Using health care use as an outcome, we only gain information on actual help seeking behavior, and we lack information on the mental health of those not seeking help. It is likely that this group consists of a mix of those not in need of help and people who are struggling, but unable to obtain adequate help. Findings using survey data support this [23]. Furthermore, we do not know the exact reasons for contacting the GP, nor do we have information on whether or what kinds of interventions were used in the consultations. To increase the chance of consultations being related to the bereavement we limited the outcome to those receiving a diagnostic code for psychological symptoms or disease. The validity of Norwegian GPs ICPC-2 coding has been deemed as good [27], but the fact that the patient presented with some kind of psychopathology, does not equate that the suicide loss was related to the patients’ problems. The statistical analyses we used take into account previous GP use, this allows us to claim with more certainty that the increase we see at the time of the death is related to the suicide loss. For the descriptive results there is more uncertainty regarding how much can be ascribed to the loss, and these numbers should be regarded as a maximum estimate of how many receive help from their GP in relation to the loss. Even if we assume that a majority of these were consultations related to the loss, we do not know if the contact was perceived as helpful. In fact, some qualitative and survey studies show that not all help from GPs in relation to suicide loss is experienced as helpful [28] and that many GPs feel unsure of how to meet the needs of bereaved patients [8, 29]. It is also important to note that other factors than degree of health issues influence help-seeking behavior. Those who are working might need to see their GP to obtain certified sick leave, while welfare beneficiaries and those who are retired who are similarly affected by the suicide might not contact their GP because they don’t have this need. Finally, we could only include the formal relations present in the official registers. Some bereaved that might be just as close to the deceased, such as close friends and romantic relations [30], are not included. We also lacked information on cohabitants that do not share their officially registered address.

Although the nature of the data used restricts the richness of our conclusions, the use of registry data covering the entire population is also the major strength of this study. By including virtually all those bereaved by suicide that can be identified in registries, the study minimizes selection bias, which is a common methodological weakness in bereavement studies [5, 31]. While there are several studies examining health care use among suicide bereaved, including specific studies on support from the GP [8], there are very few studies offering objective estimates on actual use that are not prone to selection bias. In this regard, our findings are an important contribution to the field of suicide postvention as a much-needed supplement to studies using survey and qualitative methods. This is especially important with regard to gender as the participation of men in bereavement research is difficult to obtain [24], which has led to a lack of gender balanced studies within the field [8]. The use of registries also entails a large sample size, allowing for examination of smaller subgroups and focus on different types of kinship, something that has been invited within the field [5].

Future studies should try to uncover the needs of those who have not been in contact with the health care system after suicide bereavement. Many studies have uncovered barriers to seeking help [23, 26, 32] and point out that the GP should actively reach out to those bereaved by suicide [8]. Studies examining the effect of such active outreach would be a promising avenue to pursue. The current study focused on contact with the GP, but there is also a need for more studies on total health care use to better understand the different ways those bereaved by suicide acquire help from the health care system. Preferably such studies should employ large, population representative samples to avoid selection bias. As mentioned, obtaining sick leave is probably one important need the bereaved have when they meet their GP. One study showed a tenfold increase in the risk of psychiatric sickness absence among parents who had lost an adolescent or young adult offspring to suicide [33]. Effects on occupational outcomes can have a large impact on both the individual and society, hence the need for sick leave among those bereaved by suicide should be further examined.

Our study shows that around half of those bereaved by suicide are in contact with their GP for mental health reasons following the death, and most of these are within the first months. This supports the notion that the GP is a natural place to seek help and GPs should be aware of common needs and challenges of those bereaved by suicide. At the same time, we show that there are also many that are not in contact with their GP, and this is especially true for men and bereaved siblings. Clinicians should be aware of this and consider actively reaching out to patients they know have suffered a suicide loss. Our finding that the contact rate is heightened for a relatively long period of time after the loss, and for many groups does not returns to pre-bereavement level during the study period, is useful knowledge for GPs that can be used in planning care and communicated to those bereaved to help destigmatize the need of long-term professional support after suicide bereavement. Better care for those bereaved by suicide is a goal in the Norwegian action plan for suicide prevention [34]. This paper provides important background information on one aspect of the health care use of those bereaved by suicide that is useful for policy makers going forward. The findings indicate a need to think about how to make systemic changes so that those with an under-usage can be reached, and the special needs of men and bereaved siblings should inform this work.

### Supplementary Information

Below is the link to the electronic supplementary material.

Supplementary file 1 (DOCX 145 KB)

## Data Availability

The data used in this research was obtained from a third party and are not publicly available. The researchers received the data from the registry holders as deidentified data files. The data is available on request to the registry holders, given legal and ethical approval.
