# Supplementary material for: General practitioner consultations for mental health reasons prior to and following bereavement by suicide
Source: Soc Psychiatry Psychiatr Epidemiol. 2024 Feb 6;59(9):1533–41. doi: 10.1007/s00127-023-02607-9 (PMC11343791; doi:10.1007/s00127-023-02607-9)

**Table S1** Effects of time from bereavement on monthly number of GP mental health consultations by cause of death

|  | **SUICIDE** | | | | **EXTERNAL** | | | | **OTHER** | | | |
| --- | --- | --- | --- | --- | --- | --- | --- | --- | --- | --- | --- | --- |
| **t** | ***b*** | ***p*** | **95% CI** | | ***b*** | ***p*** | **95% CI** | | ***b*** | ***p*** | **95% CI** | |
| **-24** | 0 (reference) | | | | 0 (reference) | | | | 0 (reference) | | | |
| **-23** | .000 | .921 | -.004 | .004 | .001 | .336 | -.001 | .004 | .000 | .375 | .000 | .001 |
| **-22** | .001 | .672 | -.003 | .005 | .001 | .337 | -.001 | .004 | .000 | .708 | .000 | .001 |
| **-21** | -.001 | .512 | -.006 | .003 | .001 | .270 | -.001 | .004 | .000 | .328 | .000 | .001 |
| **-20** | -.003 | .154 | -.007 | .001 | .000 | .720 | -.003 | .002 | .000 | .171 | .000 | .001 |
| **-19** | -.001 | .540 | -.006 | .003 | .000 | .825 | -.002 | .003 | .001 | .001 | .000 | .001 |
| **-18** | -.001 | .581 | -.006 | .003 | .001 | .423 | -.002 | .004 | .001 | .019 | .000 | .001 |
| **-17** | .000 | .927 | -.005 | .004 | .000 | .842 | -.002 | .003 | .001 | .007 | .000 | .001 |
| **-16** | -.003 | .246 | -.007 | .002 | .001 | .524 | -.002 | .004 | .001 | .011 | .000 | .001 |
| **-15** | .000 | .912 | -.005 | .004 | -.001 | .695 | -.003 | .002 | .001 | .001 | .000 | .001 |
| **-14** | .001 | .643 | -.004 | .006 | .001 | .486 | -.002 | .004 | .001 | .000 | .001 | .002 |
| **-13** | .002 | .454 | -.003 | .007 | .003 | .038 | .000 | .006 | .001 | .000 | .001 | .002 |
| **-12** | .003 | .236 | -.002 | .008 | .001 | .683 | -.002 | .003 | .002 | .000 | .001 | .002 |
| **-11** | .003 | .261 | -.002 | .007 | .002 | .111 | -.001 | .005 | .002 | .000 | .001 | .002 |
| **-10** | .003 | .159 | -.001 | .008 | .002 | .194 | -.001 | .005 | .002 | .000 | .001 | .002 |
| **-9** | .004 | .072 | .000 | .009 | .000 | .912 | -.003 | .003 | .002 | .000 | .002 | .003 |
| **-8** | .003 | .161 | -.001 | .008 | .001 | .307 | -.001 | .004 | .002 | .000 | .002 | .003 |
| **-7** | .004 | .113 | -.001 | .009 | .002 | .179 | -.001 | .005 | .003 | .000 | .002 | .004 |
| **-6** | .001 | .827 | -.004 | .005 | .003 | .022 | .000 | .006 | .003 | .000 | .003 | .004 |
| **-5** | .000 | .936 | -.005 | .005 | .002 | .135 | -.001 | .005 | .004 | .000 | .004 | .005 |
| **-4** | .002 | .407 | -.003 | .007 | .002 | .201 | -.001 | .005 | .005 | .000 | .004 | .006 |
| **-3** | .005 | .032 | .000 | .010 | .002 | .116 | -.001 | .005 | .007 | .000 | .006 | .007 |
| **-2** | .006 | .014 | .001 | .011 | .001 | .484 | -.002 | .004 | .010 | .000 | .009 | .010 |
| **-1** | .008 | .001 | .003 | .013 | .005 | .001 | .002 | .008 | .018 | .000 | .017 | .018 |
| **0** | .263 | .000 | .254 | .271 | .125 | .000 | .121 | .130 | .075 | .000 | .074 | .076 |
| **1** | .252 | .000 | .243 | .261 | .110 | .000 | .105 | .114 | .044 | .000 | .043 | .044 |
| **2** | .148 | .000 | .141 | .155 | .063 | .000 | .059 | .067 | .022 | .000 | .021 | .023 |
| **3** | .113 | .000 | .107 | .120 | .049 | .000 | .045 | .053 | .016 | .000 | .015 | .017 |
| **4** | .093 | .000 | .087 | .100 | .04 | .000 | .036 | .043 | .013 | .000 | .012 | .014 |
| **5** | .080 | .000 | .074 | .087 | .034 | .000 | .031 | .038 | .011 | .000 | .010 | .011 |
| **6** | .070 | .000 | .064 | .076 | .031 | .000 | .027 | .034 | .009 | .000 | .009 | .010 |
| **7** | .062 | .000 | .056 | .068 | .026 | .000 | .023 | .029 | .008 | .000 | .007 | .008 |
| **8** | .057 | .000 | .051 | .062 | .027 | .000 | .023 | .030 | .007 | .000 | .007 | .008 |
| **9** | .049 | .000 | .043 | .055 | .022 | .000 | .019 | .025 | .006 | .000 | .006 | .007 |
| **10** | .045 | .000 | .040 | .051 | .021 | .000 | .018 | .024 | .006 | .000 | .005 | .006 |
| **11** | .044 | .000 | .038 | .050 | .019 | .000 | .016 | .022 | .005 | .000 | .004 | .005 |
| **12** | .037 | .000 | .031 | .042 | .015 | .000 | .012 | .019 | .004 | .000 | .004 | .005 |
| **13** | .027 | .000 | .022 | .032 | .012 | .000 | .009 | .015 | .004 | .000 | .003 | .004 |
| **14** | .022 | .000 | .016 | .027 | .009 | .000 | .006 | .012 | .003 | .000 | .003 | .004 |
| **15** | .021 | .000 | .016 | .027 | .009 | .000 | .006 | .013 | .003 | .000 | .002 | .003 |
| **16** | .021 | .000 | .016 | .027 | .009 | .000 | .006 | .012 | .003 | .000 | .002 | .003 |
| **17** | .019 | .000 | .014 | .025 | .008 | .000 | .005 | .011 | .003 | .000 | .002 | .003 |
| **18** | .016 | .000 | .010 | .021 | .006 | .000 | .003 | .009 | .003 | .000 | .002 | .003 |
| **19** | .016 | .000 | .011 | .022 | .008 | .000 | .005 | .011 | .002 | .000 | .002 | .003 |
| **20** | .019 | .000 | .014 | .025 | .006 | .000 | .003 | .010 | .002 | .000 | .002 | .003 |
| **21** | .017 | .000 | .011 | .022 | .005 | .002 | .002 | .008 | .002 | .000 | .001 | .002 |
| **22** | .014 | .000 | .009 | .020 | .006 | .000 | .003 | .009 | .002 | .000 | .001 | .003 |
| **23** | .014 | .000 | .008 | .019 | .004 | .012 | .001 | .007 | .002 | .000 | .001 | .003 |
| **24** | .016 | .000 | .010 | .021 | .003 | .057 | .000 | .006 | .002 | .000 | .001 | .002 |

*Note.* Model estimates (*b*) at time from bereavement in months (t), p-value (*p*) and 95% confidence intervals (95% CI). Estimates are controlled for age, period, and individual fixed effects.

**Table S2** Effects of time from bereavement by suicide on monthly number of GP mental health consultations by gender

|  | **WOMEN** | | | | **MEN** | | | |
| --- | --- | --- | --- | --- | --- | --- | --- | --- |
| **t** | ***b*** | ***p*** | **95% CI** | | ***b*** | ***p*** | **95% CI** | |
| **-24** | 0 (reference) | | | | 0 (reference) | | | |
| **-23** | .001 | .666 | -.004 | .007 | -.001 | .630 | -.006 | .004 |
| **-22** | -.001 | .682 | -.007 | .005 | .004 | .181 | -.002 | .010 |
| **-21** | -.005 | .123 | -.011 | .001 | .003 | .299 | -.003 | .009 |
| **-20** | -.005 | .086 | -.011 | .001 | .000 | .966 | -.006 | .006 |
| **-19** | -.005 | .095 | -.011 | .001 | .004 | .201 | -.002 | .010 |
| **-18** | -.004 | .192 | -.011 | .002 | .003 | .363 | -.003 | .009 |
| **-17** | -.003 | .361 | -.009 | .003 | .004 | .252 | -.003 | .0100 |
| **-16** | -.004 | .271 | -.010 | .003 | -.001 | .642 | -.008 | .005 |
| **-15** | -.001 | .679 | -.008 | .005 | .001 | .684 | -.005 | .007 |
| **-14** | .000 | .897 | -.007 | .006 | .003 | .334 | -.003 | .010 |
| **-13** | .000 | .980 | -.007 | .007 | .004 | .218 | -.002 | .011 |
| **-12** | .001 | .710 | -.006 | .008 | .005 | .131 | -.001 | .011 |
| **-11** | .000 | .918 | -.006 | .007 | .006 | .084 | -.001 | .012 |
| **-10** | .003 | .406 | -.004 | .010 | .004 | .216 | -.002 | .011 |
| **-9** | .006 | .091 | -.001 | .013 | .002 | .492 | -.004 | .009 |
| **-8** | .002 | .515 | -.004 | .009 | .005 | .147 | -.002 | .012 |
| **-7** | .005 | .157 | -.002 | .012 | .002 | .481 | -.004 | .009 |
| **-6** | -.001 | .760 | -.008 | .006 | .003 | .438 | -.004 | .009 |
| **-5** | -.001 | .755 | -.008 | .006 | .002 | .583 | -.005 | .008 |
| **-4** | .002 | .575 | -.005 | .009 | .002 | .553 | -.004 | .008 |
| **-3** | .005 | .159 | -.002 | .012 | .006 | .093 | -.001 | .012 |
| **-2** | .008 | .020 | .001 | .015 | .003 | .374 | -.003 | .009 |
| **-1** | .009 | .010 | .002 | .016 | .007 | .044 | .000 | .013 |
| **0** | .317 | .000 | .305 | .329 | .189 | .000 | .178 | .201 |
| **1** | .322 | .000 | .309 | .335 | .157 | .000 | .146 | .168 |
| **2** | .198 | .000 | .187 | .208 | .081 | .000 | .072 | .090 |
| **3** | .154 | .000 | .144 | .164 | .059 | .000 | .050 | .067 |
| **4** | .128 | .000 | .119 | .138 | .046 | .000 | .038 | .054 |
| **5** | .110 | .000 | .101 | .119 | .041 | .000 | .033 | .049 |
| **6** | .098 | .000 | .089 | .107 | .033 | .000 | .025 | .040 |
| **7** | .086 | .000 | .078 | .095 | .030 | .000 | .022 | .037 |
| **8** | .076 | .000 | .068 | .085 | .030 | .000 | .022 | .038 |
| **9** | .067 | .000 | .058 | .075 | .025 | .000 | .018 | .032 |
| **10** | .059 | .000 | .051 | .068 | .026 | .000 | .018 | .033 |
| **11** | .058 | .000 | .050 | .067 | .025 | .000 | .017 | .032 |
| **12** | .050 | .000 | .042 | .058 | .019 | .000 | .012 | .027 |
| **13** | .033 | .000 | .025 | .041 | .018 | .000 | .011 | .025 |
| **14** | .027 | .000 | .019 | .034 | .014 | .000 | .007 | .021 |
| **15** | .025 | .000 | .018 | .033 | .016 | .000 | .008 | .023 |
| **16** | .025 | .000 | .017 | .033 | .016 | .000 | .009 | .023 |
| **17** | .023 | .000 | .015 | .030 | .015 | .000 | .007 | .022 |
| **18** | .016 | .000 | .009 | .024 | .014 | .000 | .007 | .021 |
| **19** | .022 | .000 | .014 | .030 | .009 | .014 | .002 | .016 |
| **20** | .021 | .000 | .013 | .029 | .017 | .000 | .009 | .024 |
| **21** | .020 | .000 | .012 | .028 | .012 | .001 | .005 | .019 |
| **22** | .016 | .000 | .008 | .023 | .012 | .001 | .005 | .019 |
| **23** | .017 | .000 | .009 | .025 | .009 | .012 | .002 | .016 |
| **24** | .019 | .000 | .011 | .027 | .011 | .003 | .004 | .018 |

*Note.* Model estimates (*b*) at time from bereavement in months (t), p-value (*p*) and 95% confidence intervals (95% CI). Estimates are controlled for age, period, and individual fixed effects.

**Table S3** Effects of time from bereavement by suicide on monthly number of GP mental health consultations by kinship

|  | **PARTNER** | | | | **OFFSPRING** | | | | **PARENT** | | | | **SIBLING** | | | |
| --- | --- | --- | --- | --- | --- | --- | --- | --- | --- | --- | --- | --- | --- | --- | --- | --- |
| **t** | ***b*** | ***p*** | **95% CI** | | ***b*** | ***p*** | **95% CI** | | ***b*** | ***p*** | **95% CI** | | ***b*** | ***p*** | **95% CI** | |
| **-24** | 0 (reference) | | | | 0 (reference) | | | | 0 (reference) | | | | 0 (reference) | | | |
| **-23** | .007 | .262 | -.005 | .019 | -.005 | .259 | -.013 | .004 | .009 | .029 | .001 | .017 | -.004 | .186 | -.010 | .002 |
| **-22** | .003 | .674 | -.010 | .015 | -.008 | .050 | -.017 | .000 | .007 | .129 | -.002 | .015 | .002 | .479 | -.004 | .009 |
| **-21** | .010 | .126 | -.003 | .022 | -.009 | .062 | -.018 | .000 | .003 | .523 | -.006 | .012 | -.003 | .400 | -.010 | .004 |
| **-20** | .005 | .469 | -.008 | .018 | -.012 | .012 | -.021 | -.003 | -.004 | .396 | -.012 | .005 | .000 | .983 | -.007 | .007 |
| **-19** | .001 | .829 | -.010 | .013 | -.006 | .197 | -.015 | .003 | -.001 | .735 | -.010 | .007 | .001 | .867 | -.006 | .008 |
| **-18** | .002 | .696 | -.010 | .015 | -.008 | .120 | -.017 | .002 | -.002 | .665 | -.010 | .007 | .002 | .634 | -.005 | .009 |
| **-17** | .003 | .618 | -.009 | .016 | -.009 | .077 | -.018 | .001 | .004 | .396 | -.005 | .013 | .001 | .751 | -.006 | .008 |
| **-16** | .005 | .418 | -.008 | .018 | -.008 | .129 | -.018 | .002 | .001 | .814 | -.008 | .010 | -.004 | .215 | -.011 | .003 |
| **-15** | .004 | .544 | -.010 | .019 | -.004 | .399 | -.014 | .006 | .000 | .913 | -.008 | .009 | .000 | .962 | -.007 | .007 |
| **-14** | .005 | .485 | -.009 | .019 | -.007 | .164 | -.018 | .003 | .009 | .062 | .000 | .018 | .000 | .991 | -.007 | .007 |
| **-13** | .013 | .081 | -.002 | .028 | -.007 | .210 | -.017 | .004 | .004 | .379 | -.005 | .013 | .002 | .580 | -.005 | .009 |
| **-12** | .006 | .408 | -.009 | .021 | -.004 | .480 | -.014 | .007 | .004 | .400 | -.005 | .013 | .005 | .183 | -.002 | .012 |
| **-11** | .015 | .051 | .000 | .030 | -.006 | .289 | -.016 | .005 | .004 | .387 | -.005 | .013 | .003 | .396 | -.004 | .010 |
| **-10** | .015 | .062 | -.001 | .030 | -.004 | .420 | -.015 | .006 | .006 | .212 | -.003 | .015 | .003 | .414 | -.004 | .010 |
| **-9** | .013 | .105 | -.003 | .028 | -.004 | .455 | -.015 | .007 | .013 | .011 | .003 | .022 | .002 | .612 | -.005 | .009 |
| **-8** | .020 | .011 | .005 | .035 | -.008 | .110 | -.018 | .002 | .007 | .174 | -.003 | .016 | .003 | .360 | -.004 | .011 |
| **-7** | .028 | .001 | .011 | .044 | -.002 | .662 | -.013 | .008 | .007 | .149 | -.002 | .016 | -.001 | .760 | -.008 | .006 |
| **-6** | .014 | .073 | -.001 | .030 | -.003 | .546 | -.014 | .007 | .003 | .497 | -.006 | .013 | -.003 | .428 | -.010 | .004 |
| **-5** | .031 | .000 | .014 | .047 | -.003 | .597 | -.013 | .007 | -.001 | .763 | -.011 | .008 | -.006 | .117 | -.013 | .001 |
| **-4** | .039 | .000 | .023 | .056 | -.009 | .081 | -.019 | .001 | .008 | .095 | -.001 | .018 | -.006 | .087 | -.013 | .001 |
| **-3** | .026 | .001 | .010 | .042 | -.003 | .531 | -.014 | .007 | .011 | .025 | .001 | .020 | .001 | .834 | -.006 | .008 |
| **-2** | .041 | .000 | .024 | .058 | .000 | .982 | -.011 | .010 | .010 | .027 | .001 | .020 | -.003 | .384 | -.010 | .004 |
| **-1** | .057 | .000 | .039 | .074 | -.001 | .797 | -.012 | .009 | .010 | .031 | .001 | .019 | -.001 | .747 | -.008 | .006 |
| **0** | .503 | .000 | .470 | .535 | .300 | .000 | .281 | .320 | .261 | .000 | .245 | .277 | .178 | .000 | .166 | .189 |
| **1** | .481 | .000 | .449 | .513 | .346 | .000 | .325 | .366 | .216 | .000 | .199 | .232 | .161 | .000 | .149 | .173 |
| **2** | .33 | .000 | .304 | .357 | .235 | .000 | .218 | .252 | .109 | .000 | .096 | .122 | .076 | .000 | .066 | .085 |
| **3** | .268 | .000 | .243 | .292 | .194 | .000 | .178 | .211 | .072 | .000 | .060 | .084 | .053 | .000 | .044 | .062 |
| **4** | .221 | .000 | .198 | .244 | .165 | .000 | .150 | .181 | .063 | .000 | .051 | .074 | .038 | .000 | .030 | .047 |
| **5** | .198 | .000 | .176 | .221 | .140 | .000 | .125 | .155 | .051 | .000 | .040 | .062 | .034 | .000 | .026 | .042 |
| **6** | .166 | .000 | .146 | .187 | .127 | .000 | .112 | .141 | .043 | .000 | .033 | .054 | .030 | .000 | .022 | .038 |
| **7** | .160 | .000 | .139 | .181 | .109 | .000 | .094 | .123 | .035 | .000 | .025 | .046 | .027 | .000 | .019 | .035 |
| **8** | .138 | .000 | .118 | .158 | .090 | .000 | .076 | .103 | .039 | .000 | .029 | .050 | .027 | .000 | .019 | .036 |
| **9** | .131 | .000 | .111 | .151 | .086 | .000 | .073 | .100 | .031 | .000 | .020 | .041 | .018 | .000 | .010 | .026 |
| **10** | .120 | .000 | .100 | .140 | .078 | .000 | .065 | .092 | .031 | .000 | .020 | .041 | .016 | .000 | .008 | .024 |
| **11** | .104 | .000 | .085 | .123 | .069 | .000 | .055 | .082 | .027 | .000 | .017 | .038 | .025 | .000 | .016 | .033 |
| **12** | .099 | .000 | .080 | .118 | .058 | .000 | .044 | .071 | .030 | .000 | .020 | .041 | .013 | .002 | .005 | .021 |
| **13** | .062 | .000 | .045 | .079 | .044 | .000 | .031 | .056 | .025 | .000 | .015 | .035 | .009 | .024 | .001 | .017 |
| **14** | .054 | .000 | .036 | .072 | .025 | .000 | .014 | .037 | .022 | .000 | .012 | .032 | .010 | .013 | .002 | .018 |
| **15** | .045 | .000 | .028 | .062 | .029 | .000 | .016 | .041 | .023 | .000 | .013 | .033 | .009 | .020 | .001 | .017 |
| **16** | .044 | .000 | .027 | .061 | .032 | .000 | .019 | .045 | .022 | .000 | .012 | .033 | .008 | .038 | .000 | .016 |
| **17** | .043 | .000 | .026 | .061 | .022 | .001 | .009 | .035 | .021 | .000 | .010 | .032 | .010 | .016 | .002 | .018 |
| **18** | .040 | .000 | .023 | .058 | .019 | .002 | .007 | .031 | .017 | .002 | .006 | .027 | .006 | .154 | -.002 | .014 |
| **19** | .035 | .000 | .018 | .051 | .017 | .007 | .005 | .029 | .016 | .002 | .006 | .026 | .011 | .008 | .003 | .019 |
| **20** | .035 | .000 | .018 | .052 | .027 | .000 | .015 | .040 | .019 | .001 | .008 | .029 | .010 | .014 | .002 | .018 |
| **21** | .046 | .000 | .028 | .065 | .021 | .001 | .008 | .033 | .013 | .012 | .003 | .024 | .008 | .048 | .000 | .017 |
| **22** | .032 | .000 | .015 | .048 | .015 | .02 | .002 | .027 | .015 | .005 | .005 | .025 | .008 | .048 | .000 | .016 |
| **23** | .029 | .000 | .013 | .044 | .014 | .022 | .002 | .027 | .014 | .006 | .004 | .025 | .008 | .049 | .000 | .016 |
| **24** | .024 | .005 | .007 | .041 | .020 | .002 | .007 | .033 | .013 | .014 | .003 | .023 | .012 | .006 | .003 | .020 |

*Note.* Model estimates (*b*) at time from bereavement in months (t), p-value (*p*) and 95% confidence intervals (95% CI). Estimates are controlled for age, period, and individual fixed effects.

**Figure S1** Most common ICPC-2 psychological codes in consultations with a GP within the first year after bereavement from suicide deaths, external deaths and other deaths


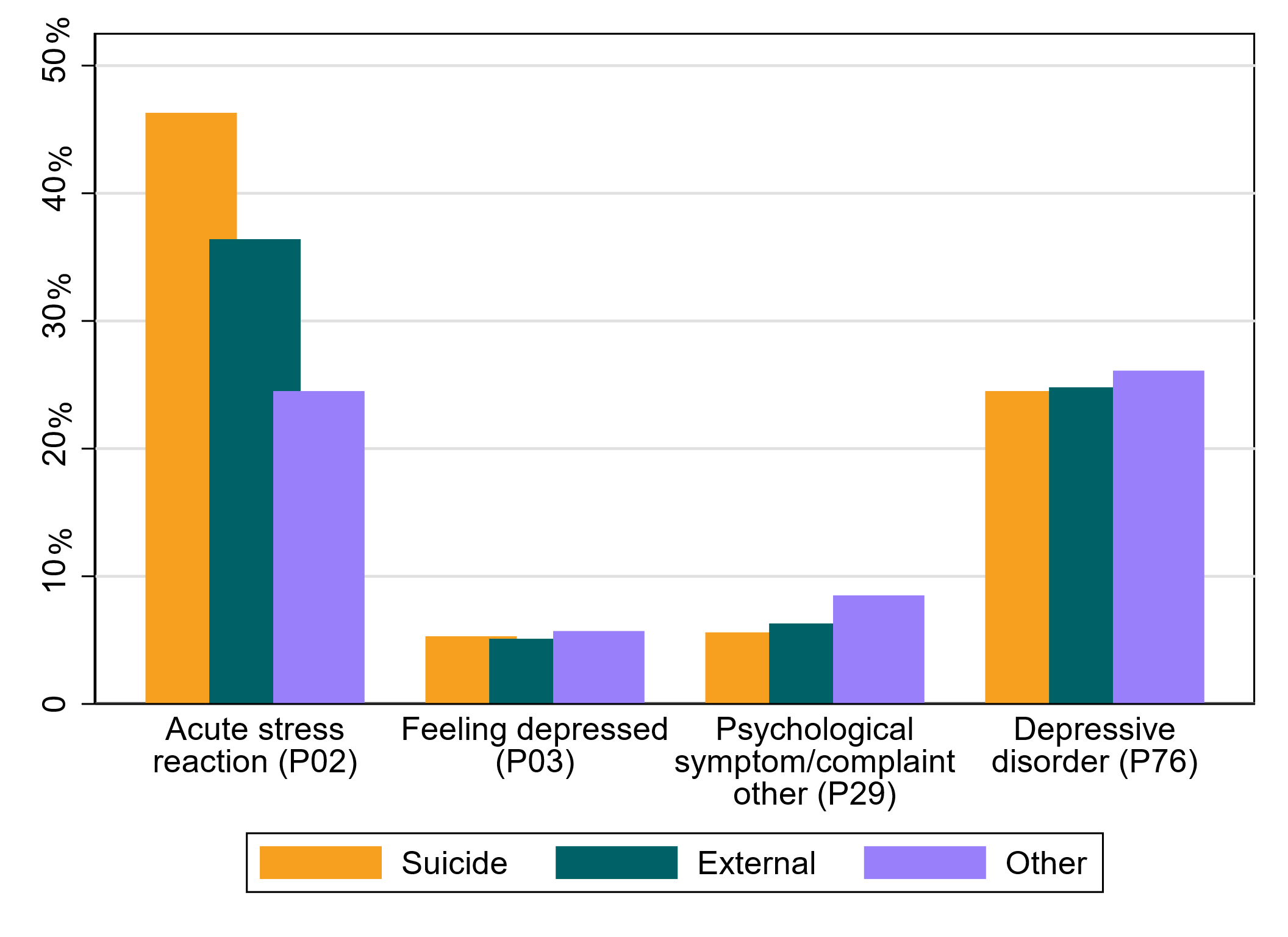

Supplement: Supplementary file 1 — (DOCX 145 KB) [file 127_2023_2607_MOESM1_ESM.docx]
